# Yawning Detection Sensitivity and Yawning Contagion

**DOI:** 10.1177/2041669517726797

**Published:** 2017-08-25

**Authors:** Meingold H. M. Chan, Chia-Huei Tseng

**Affiliations:** Department of Psychology, University of Cambridge, UK; Research Institute of Electrical Communication, Tohoku University, Sendai, Japan

**Keywords:** contagious yawning, perceptual sensitivity, face perception, eye gazing, autistic trait, individual difference, emotion

## Abstract

Contagious yawning—the urge to yawn when thinking about, listening to, or viewing yawning—is a well-documented phenomenon in humans and animals. The reduced yawn contagion observed in the autistic population suggested that it might be empathy related; however, it is unknown whether such a connection applies to nonclinical populations. We examined influences from both empathy (i.e., autistic traits) and nonempathy factors (i.e., individuals’ perceptual detection sensitivity to yawning, happy, and angry faces) on 41 nonclinical adults. We induced contagious yawning with a 5-minute video and 20 yawning photo stimuli. In addition, we measured participants’ autistic traits (with the autism-spectrum quotient questionnaire), eye gaze patterns, and their perceptual thresholds to detect yawning and emotion in human face photos. We found two factors associated with yawning contagion: (a) those more sensitive to detect yawning, but not other emotional expressions, displayed more contagious yawning than those less sensitive to yawning expressions, and (b) female participants exhibited significantly more contagious yawning than male participants. We did not find an association between autistic trait and contagious yawning. Our study offers a working hypothesis for future studies, in that perceptual encoding of yawning interacts with susceptibility to contagious yawning.

## Introduction

Contagious yawning, the urge to yawn when thinking about, listening to, or viewing yawning (Baenninger, 1987), is a well-documented phenomenon observed in human beings, primates (Anderson, Myowa-Yamakoshi, & Matsuzawa, 2004), and dogs (Harr, Gilbert, & Phillips, 2009). Despite research efforts across more than three decades, the underlying mechanism of contagious yawning remains unclear. However, an expanding range of hypotheses have been proposed, including an innate releasing mechanism (Provine, 1986, 1989), respiratory or circulatory, thermoregulation, the arousal hypothesis, and the social communication view (for a review, see Guggisberg, Mathis, Schnider, & Hess, 2010). Among them, the most intriguing hypothesis that has aroused many researchers’ interest is the link between empathy and contagious yawning. For instance, Platek, Critton, Myers, and Gallup (2003) found that contagious yawning could be understood as a social behavior that involves mental attribution (i.e., the propensity to understand another’s mental state). They found that individuals with higher susceptibility to contagious yawning are better at recognizing their own faces (i.e., self-face recognition) and theory of mind tasks that capture one’s social understanding.

If contagious yawning is indeed a social behavior, what is its purpose? Guggisberg and coworkers (2010) attempted to explain the social function of contagious yawning by proposing yawning as a communication signal that spread to other people for survival purposes (i.e., the social or communication hypothesis of yawning). While the biological foundation of yawning might be a change in physiological state (e.g., lung oxygen levels decrease or brain temperature increases) in response to our environment, they proposed that the contagious effect of yawning allowed us to communicate with our social group and promotes behavioral synchronization for facing potential threat. Indeed, this idea is not entirely novel—in an earlier study, Provine (1989) stated that “the chain reaction of contagious yawning synchronizes the physiological as well as behavioral state of the group” (p. 213). Similarly, Brothers (1990) introduced the idea that yawning could be a form of social signaling akin to laughter that is contagious. Although speculative, the social or communication hypothesis helps explain the social function of contagious yawning and suggests an account for the relationship between contagious yawning and social understanding.

Neuroimaging studies have found that brain regions associated with contagious yawning are also related to social understanding and empathy. Activation in the ventromedial prefrontal cortex, which is known for the processing of social cues and recognition of complex emotional expression (Eslinger & Damasio, 1985; Wager, Phan, Liberzon, & Taylor, 2003), increased during free viewing of a yawning video but not when the video stimuli displayed gaping, coughing, or expressionless faces (Nahab, Hattori, Saad, & Hallet, 2009). Schürmann et al. (2005) showed that BOLD response signals in the superior temporal sulcus, a region known for being involved in facial expression recognition and empathy (see Narumoto, Okada, Sadato, Fukui, & Yonekura, 2001; Calder & Young, 2005, for a review), increased significantly when participants watched yawning videos, but not mouth opening videos, and was positively associated with the urge to yawn. Platek, Mohamed, and Gallup (2005) also revealed that watching yawning videos evoked unique activation in the posterior cingulate cortex and precuneus, which play important roles in theory of mind and social processing (Blakemore, Rees, & Frith, 1998; Fletcher et al., 1995). These studies together suggest that brain regions involved in social processing may be heavily involved in contagious yawning.

Clinical reports also suggest that individuals’ social understanding may relate to their susceptibility to contagious yawning. Children aged 7 to 15 years who had been diagnosed with an autism spectrum disorder (ASD)—a range of developmental disorders characterized by deficits in social interaction—showed reduced contagious yawning compared with typically developing (TD) individuals when watching a yawning video but not when watching a smiling video (Senju et al., 2007). Helt, Eigsti, Snyder, and Fein (2010) matched mental ages between ASD and TD children aged 5 to 12 years and replicated the finding that children with ASD were significantly less likely than TD children to yawn after being exposed to the experimenter’s yawn. In addition, Giganti and Esposito Ziello (2009) compared the frequency of contagious yawning between ASD children with varied severity (characterized by the Childhood Autism Rating Scale) and found that children with low autistic severity elicited more contagious yawning than those with high autistic severity. The clinical reports signified a possible relationship between autistic characteristics and susceptibility to contagious yawning (Baron-Cohen, Leslie, & Frith, 1985; Platek et al., 2003). Nonetheless, it is unclear whether this connection applies to a nonautistic population that can be viewed as a continuous spectrum or exists only in the autistic population.

Previous studies have indicated that even among nonautistic populations, not all individuals were susceptible to contagious yawning. Only 40% to 60% of the nonautistic population display contagious yawning in response to a yawn stimulus (Hoogenhout, van der Straaten, Pileggi, & Malcolm-Smith, 2013; Platek et al., 2003), indicating individual variability in susceptibility to contagious yawning. However, little is known about factors that determine such individual differences. To our best knowledge, only one recent study assessed a comprehensive range of factors in explaining individual variation in susceptibility to contagious yawning (Bartholomew & Cirulli, 2014). Among all variables (i.e., basic demographics, empathy, sleep, cognitive performance, testing conditions, and time of day), age, not empathy, was the only factor that could significantly predict individual variation in susceptibility to contagious yawning: Older participants yawned less than younger participants. Yet, age only explained 8% of the variation, which leaves a huge amount of variation unexplained. Therefore, studies of other variables beyond the commonly studied social factors (e.g., empathy) are warranted.

Apart from social difficulties, one well-known characteristic of autistic people is their atypical eye-gazing pattern during face processing, that is, looking at the mouth more than the eyes (Blair, 2005; Gepner, Gelder, & Schonen, 1996; Golarai, Grill-Spector, & Reiss, 2006). This perceptual distinction was proposed as an alternative account for the notable social deficits in the ASD population: Their proclivity for not attending to regions containing the most social information (e.g., eyes) might create a perceptual bottleneck for the subsequent processing of social interactions (Baron-Cohen, Wheelwright, & Jolliffe, 1997; Dawson et al., 2004).

Interestingly, two recent findings indicated that directing attention to the eyes could effectively restore susceptibility to contagious yawning in autistic individuals to the same extent as nonautistic individuals. Senju et al. (2009) instructed both participants with and without autism to fixate on a location where the eyes of face stimuli would appear. The participants were asked to count the number of female faces while watching video clips of yawning or control mouth movements. When autistic individuals directed their attention to the eyes, they displayed equally frequent yawning responses toward yawning stimuli as did nonautistic individuals. Yet, this study lacked objective measurement (e.g., an eye tracker) of the actual eye gaze to validate whether the effect was truly from gaze redirection. With the assistance of an eye tracker, Usui et al. (2013) initiated yawning or control videos only after participating ASD children had continuously fixated on the eye region of the actor for 500 ms. In the task, participating children counted the number of actors wearing glasses, and Usui et al. (2013) replicated the results from Senju et al.’s (2009) study. The importance of access to the eye region of an inducing stimulus was reported in nonclinical participants (Provine, 1989). When participants viewed a variety of yawning stimuli including a complete-face yawn, no-mouth yawn, no-eyes yawn, and a control stimulus (smile), complete-face yawns evoked significantly more yawners than a control smiling face (Provine, 1989). The “no-mouth” yawn was the only stimuli with deleted facial features that generated as many yawners as the complete-face yawn. Conceivably, removing facial features of a yawn increases the detection difficulty. This in turn diminishes the contagion effect of yawning and evokes fewer yawners. This finding also suggests that the eye region may contain more fundamental information to evoke contagious yawning than the mouth region.

Other than experimental manipulation to increase detection difficulty by reducing accessibility of facial parts to viewers, does an individual’s inherent detection sensitivity to a yawning expression preclude his or her susceptibility to contagious yawning? This question is currently unanswered. Similar to contagious yawning, emotional contagion is well documented as a highly unconscious and automatic behavior of mimicking others’ emotional expression. Studies have shown that individuals especially susceptible to emotional contagion are those who can read others’ emotional expressions and are sensitive to others’ emotions (Doherty, 1997), and that those who mimic others’ expressions are better at recognizing others’ emotions (Oberman, Winkielman, & Ramachandran, 2007; Stel & van Knippenberg, 2008). Although yawning is not considered an emotion and contagious yawning is not understood as an example of emotional contagion, it is possible that a similar mechanism contributes to the individual variation of both contagion phenomena.

The contagion effect occurs not only unintentionally in higher level imitation such as action or emotional contagion but also in lower level nonemotional responses such as heart rate (Dimascio, Boyd, & Greenblatt, 1957), pupil size (del Valle Loarte & Garcia Ruiz, 2009; Harrison, Singer, Rotshtein, Dolan, & Critchley, 2006), and temperature contagion (Cooper et al., 2014). The temperature of a participant’s hand has been found to decrease significantly after s/he observed and rated the perceived temperature of actors whose hands were immersed in ice-cold water. Since one possible function of yawning is to lower our body temperature to protect us from critical brain temperature rises, it is possible that both contagious yawning and temperature contagion are important for temperature regulation (Gallup & Eldakar, 2013; Gallup & Gallup, 2008). Notably, the link between sensitivity to temperature contagion and empathy is not clear. A negative correlation between sensitivity to temperature contagion and the Mehrabian balanced emotional empathy scale (a 30-item questionnaire, including items such as “It upsets me to see someone being mistreated” rated on a 9-point agree–disagree scale that assess individual’s emotional empathy) was found. However, a positive correlation was found with the empathy concern subscale of the Davis interpersonal reactivity scale rated on a 5-point scale (7-item Empathy Concern subscale e.g., “I often have tender, concerned feelings for people less fortunate than me”). This suggests that the relationship between individual differences in empathy and low-level contagion phenomena may not be a simple one.

In this study, we contributed to the little-investigated area of individual differences in contagious yawning in a nonclinical population by observing individuals’ autistic traits, and perceptual detection sensitivity to yawning expressions. We aimed to investigate the interplay between one’s yawning detection sensitivity and contagious yawning. We first attempted to extend the clinical findings of the link between autistic traits and contagious yawning to a nonclinical sample by examining the association between autism-spectrum quotient (AQ) scores and contagious yawning. Then, we tested the association between sensitivity to emotional/yawning expressions and contagious yawning. The study of individuals’ susceptibility to contagious yawning could provide insight into psychiatric disorders such as schizophrenia and autism, as well as general human functioning related to yawning and the contagion effect. Our hypotheses are as follows:

1. Individuals with higher autistic tendency will display less contagious yawning.
2. Individuals with higher sensitivity to yawning expressions will display more contagious yawning.

## Methods

### Participants

Twenty male and 21 female Cantonese-speaking participants ranging from 19 to 26 years old (mean = 21.3, *SD* = 1.89) participated in exchange for course credits. Written informed consent was obtained from all individual participants. Although there were no exclusion criteria during the recruitment of participants, in a postexperiment interview, 34 participants (out of 41) reported that they were not taking any medication at the time of experiment and did not have any history of psychiatric disorders including autism, which could affect one’s perception and expression of one’s own and others’ emotions. We could not contact the remaining seven participants after the experiment.

### Stimuli

#### Video stimuli to induce yawning

We recruited six actors and recorded two 5-minute videos while they talked about their university lives. In one video, actors yawned 10 times in total with a gap of approximately 20 seconds in between. Each yawn lasted for 3 to 6 seconds. In the other control video, actors smiled 10 times with a gap of 20 seconds, and each smile lasted for 2 to 4 seconds.

#### Photo stimuli to induce yawning

We took screenshots of 20 actors yawning at the maximum intensity from a video aimed to induce yawning made available to the public by AsapSCIENCE online (https://youtu.be/AJXX4vF6Zh0). Half of the actors were female. Due to availability, all actors were Caucasian except for one Asian male.

#### Perceptual detection sensitivity task

We tested participants’ detection thresholds in three expression categories: happiness, anger, and yawning. We first selected stimuli that would allow us to generate a complete psychometric function to estimate the thresholds. Therefore, at one end of the spectrum were the expression that needed to be obviously present (so that participants would always report positive detection); at the other end of the spectrum were the expression that needed to be obviously absent (so that participants would never report detection). Later we describe how the tested images were selected.

#### Yawning

**Figure 1.**
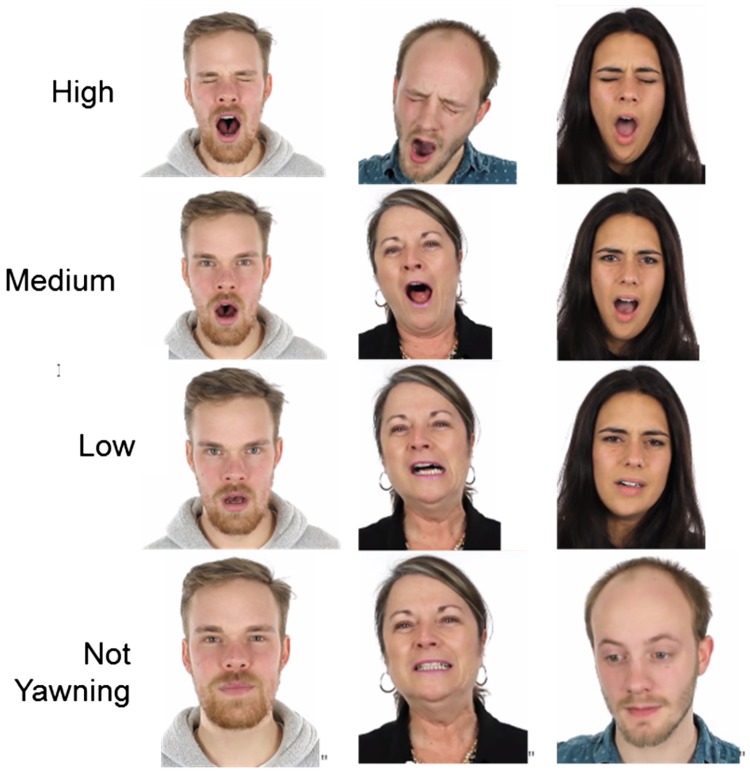
Yawning photo stimuli for perceptual detection sensitivity task.

Because there were no standardized images for yawning available, we first took screenshots of a total of 29 potential yawning photo stimuli from the same video used for creating photo stimuli to induce yawning. We took screenshots of four actors in the video who displayed gradual yawns with various intensities. All photos were then rated twice in an online questionnaire pilot study by 22 individuals (11 male) on a 5-point rating (1 = *completely disagree*, 5 = *completely agree*) to answer the question “The person in the photo is yawning.” The average rating of each photo ranged from 1.05 (*strong disagreement*) to 4.50 (*strong agreement*), mean = 3.19. Based on the average rating for each image, we classified the photos into four levels of intensity: High (4 ± 0.5), medium (3 ± 0.5), low (2 ± 0.5), and not yawning (1 ± 0.5). For easy reference, we labeled the four conditions with numbers (1 = not yawning to 4 = high). We selected three photos at each intensity level (see Figure 1). Six of the 12 photos were of females, and the other six were of males.

#### Happy

**Figure 2.**
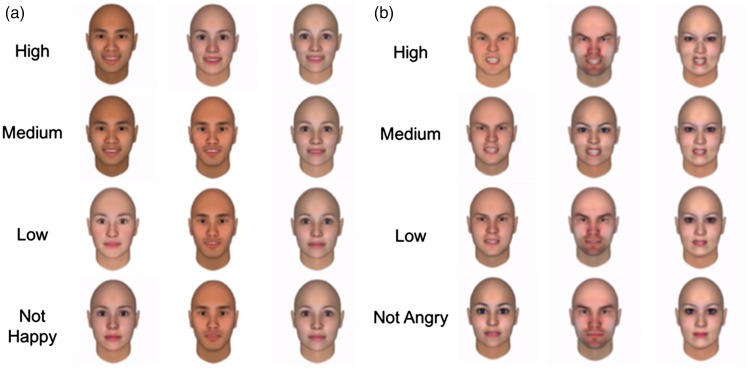
(a) Happy and (b) angry photo stimuli of different intensities used in the emotional detection task.

We used FaceGen Modeler (Singular Inversions Inc., 2010), a data-driven statistical model based on three-dimensional laser-scanned face database (Blanz & Vetter, 1999) for generated face stimuli. This allowed us to parametrically adjust along multiple dimensions including age, gender, ethnicity, and emotional expression. We chose to use FaceGen because it has been validated by human participant rating (Roesch et al., 2011) and has been widely used in studies related to emotional expression (e.g., Hass, Weston, & Lim, 2016; N’Diaye, Sander, & Vuilleumier, 2009; Oosterhof & Todoro, 2009). To prepare the final 12 face stimuli to test participants’ sensitivity to detect happy faces, we first generated 36 happy faces from four identities (two females) with FaceGen Modeler by varying expressiveness at 0%, 12%, 25%, 32%, 50%, 62%, 75%, 87%, and 100%. All faces were centered at 30 years old, and three ethnicities were selected for diversity (i.e., two European females, one East Asian male, and one South Asian male). The same group who rated the yawning photos rated these photos using the same procedure by answering the question “The person in the photo is happy.” The average rating of each photo ranged from 1.17 (*strong disagreement*) to 4.76 (*strong agreement*), mean = 3.07. We used the average rating to classify the photos into four intensity levels based on the following criteria: high (> 4.5), medium (3.5–4.5), low (2.5–3.5), and not happy (< 2.5). For easy reference, we labeled the four intensities as follows: high = 4, medium = 3, low = 2, and not happy = 1. The final selection of 12 photos (three in each intensity, six female) is shown in Figure 2(a).

#### Angry

An identical procedure was applied to produce angry face stimuli. We first generated 36 angry faces from four new identities (two females) of three ethnicities (i.e., two European male, one East Asian female, and one South Asian female) using FaceGen Modeler. The average rating of each photo ranged from 1.25 (*strong disagreement*) to 4.75 (*strong agreement*), mean = 3.09. The final selection of 12 photos (three in each intensity, half female) is shown in Figure 2(b).

### Apparatus and Procedure

The study was approved by the Research Ethics Committee at the University of Hong Kong. All participants were tested individually in a quiet room between 2 and 6 p.m. to avoid the peak hours of yawning among young adults (Zilli, Giganti, & Uga, 2008). All participants were informed that the study was on impression formation and they had to select a suitable drama character candidate from each video that fit the requirements of the character role. The cover story aimed to disguise the true purpose of this study. All participants were asked to sign an informed consent form.

**Figure 3.**
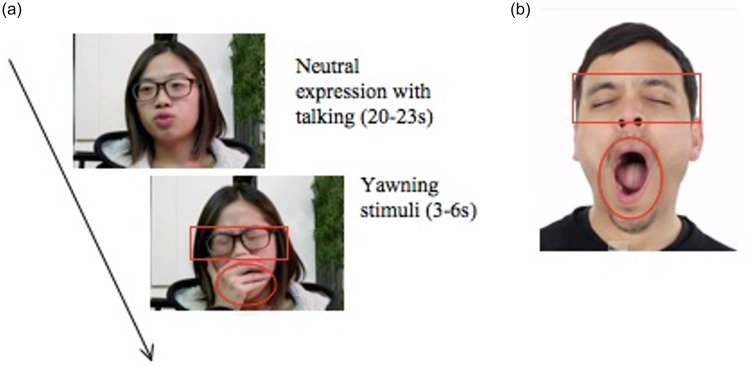
Stimuli marked with areas of interest (eyes and mouth). (a) 5-minute video stimuli alternated between neutral expression and yawning (10 alternations in total). (b) A yawning photo with the highest intensity. The eye and mouth regions of actor’s yawning were marked as area of interest (AOI; the red outlines in the figures did not appear in the real experiment).

Participants first completed the AQ questionnaire (Baron-Cohen, Wheelwright, Skinner, Martin, & Clubley, 2001) and the Perceived Arousal Scale (PAS; Nicassio, Mendlowitz, Fussell, & Petras, 1985) followed by 1 minute of music to relax. To induce yawning, participants watched one 5-minute yawning and one 5-minute control smiling video, in counterbalanced order before they viewed an additional 20 yawning photos. The 5-minute video stimuli included neutral expressions of 20 to 23 seconds alternating with 3 to 6 seconds of yawning or smiling for 10 times in total. Twenty yawning photos were shown after both yawning and control videos were watched. Each photo appeared for 5 seconds in random sequence. After viewing all photos, participants rated their urge to yawn on a 5-point scale in response to the statement “The photo made me want to yawn” (1 = *completely disagree*, 5 = *completely agree*). A Tobii T120 eye-tracking system was used to present stimuli and record observers’ eye-gazing patterns while a separate hidden webcam recorded the participants’ yawning. The area of interest (AOI) for eye-gazing pattern is the eye and mouth region of the actor (Figure 3(a) and (b)). At the end, participants were debriefed, and the purpose of the research was explained in detail. The experimenter informed participants that their yawning behavior was recorded by a hidden webcam and asked their permission for later behavioral coding. All participants granted their permission to the experimenter to use their video in addition to the consent form signed at the beginning of the experiment.

Observers’ detection sensitivity was estimated from their judgment of whether the person in a photo was yawning or not by pressing a *yes* or a *no* key. Similarly, in a separate block, their sensitivity to anger was obtained by judging whether the person in a photo was angry or not. The same applied for the sensitivity to happiness. Each expression contained 60 photos (4 intensities × 3 identities × 5 repeats). Each photo was shown for 2 seconds on a 19-in. ViewSonic G90fB monitor and displayed in random sequence. Three practice trials were given prior to the real experiment to familiarize participants with the response keys.

### Coding of Yawning

The yawning responses of participants were later encoded offline by a coder blind to the video sequences. The coder would record the presence of yawning when the subject displayed gaping of the mouth with a long inspiration and short expiration (Usui et al., 2013). Since inhibition of yawning may occur due to politeness (Provine, 2005), which was also reported by participants in the pilot study, a hint of yawning with opening of nostril without mouth opening or eyes closing was also counted as yawning. Participants were also asked to report their urge to yawn on a 5-point scale after the yawning photo stimuli, to assess their subjective feelings in addition to their overt behavior. This way of measuring “yawning susceptibility” has also been used in past studies (e.g., Schürmann et al., 2005).

## Results

Since our count data of contagious yawning are highly skewed and contained an excessive count of zeros (55%), we examined the association between contagious yawning and other variables using a Poisson regression (Hoogenhout et al., 2013), which is well suited for this kind of count data.

To facilitate cross-study comparison, we reported effect size of each statistical test. Effect size (*r*) of nonparametric test, including Mann–Whitney *U* test and Wilcoxon Signed Rank test, is calculated with the formula,

$$r=\frac{Z}{\sqrt{N}}$$

where *Z* represents *z* value of the Mann–Whitney *U* or Wilcoxon Signed Rank test, and *N* represents sample size. The effect size of Poisson regression is reported in odd ratios (labeled as e^B^; e = Exp; B = standardized coefficient).

### AQ Score

The AQ score of our participants ranged from 7 to 38 (mean = 18.34, *SD* = 6.80). In a study with individuals with ASD, 80% obtained an AQ score higher than 32 (Baron-Cohen et al., 2001). Although in our sample two participants obtained an AQ score higher than 32 (37 and 38, respectively), neither subject had been diagnosed with ASD.

An independent *t* test indicated that there was no significant gender difference in total AQ score, *p* = .493, Cohen’s *d* = 0.22. We used a nonparametric Mann–Whitney test to examine gender differences in AQ subscales as the scores in each scale were not normally distributed. All AQ subscales (i.e., communication, social skills, attention switching, attention to details, and imagination) had no significant gender differences.

### Perceived Arousal Scale

The PAS score indicates the arousal level of participants before starting the experiment (Anderson, Anderson, & Deuser, 1996; Anderson, Deuser, & DeNeve, 1995). The PAS is a scale developed to measures participants’ overall arousal with a high internal reliability (Anderson et al., 1995) and has been widely used in studies of hostile or aggressive behavior (e.g., Anderson, Carnagey, & Eubanks, 2003; Glascock, 2015; Ivory & Kaestle, 2013). Participants rated 24 adjectives on a 5-point scale including 10 adjectives reflecting high arousal and 14 adjectives reflecting low arousal. A total arousal score is derived by reverse scoring the low arousal subscale and summing the high arousal subscale score with the low arousal subscale score. Thus, a higher total score indicates higher arousal. The possible range of the score is 24 (lowest arousal) to 120 (highest arousal). The PA score of our participants ranged from 53 to 107 (mean = 82.92, *SD* = 15.60), which is similar to the baseline condition average (86.1) in Anderson et al. (1995). The PA score was not associated with the counts of yawning, Poisson regression: χ^2^(1, *N* = 40)=0.00, *p* = .99, e^B^ ranged from .99 to 7.22E+11, or urge to yawn (Spearman’s correlation: *r_s_* = −.15, *p* = .40).

### Contagious Behavior

We examined the participants’ yawning behavior. Nineteen of the 41 participants (46.3%) yawned contagiously at least once in either the yawning video or the photo section, which fell into the range reported in previous studies (i.e., 45%–60%; Hoogenhout et al., 2013; Platek et al., 2003). The average frequency of contagious yawning obtained in both the yawning video and photo stimuli was 0.91 (*SD* = 1.80, max = 10). Of the participants, 45% reported that the yawning stimuli made them want to yawn (a score of 4 or above on a 5-point scale), similar to a past study that used the same measure of yawning susceptibility (i.e., 40%; Nahab et al., 2009). The median score of urge to yawn reported after watching all the yawning stimuli was 3 out of 5.

One subject was excluded from the analysis as her contagious yawning frequency exceeded 2 *SD* (i.e., an outlier). Therefore, data of 40 participants were entered into the final analysis. All statistical analyses were completed using the Statistical Package for the Social Sciences (SPSS, 2012).

### Yawning-Induction Stimuli Validation

To validate our yawning-induction stimuli, we conducted a nonparametric Wilcoxon Signed Ranks test to compare the frequency of yawning during the presentation of control versus yawning stimuli. The frequency of yawning during the presentation of yawning stimuli, *Mdn* = 9.50, was significantly higher than during the presentation of control stimuli, *Mdn* = 0, *Z* = −3.85, *p* < .001, *r* = −0.61.

Furthermore, a difference in yawning behavior was observed between the two types of yawning stimuli (i.e., video vs. photo stimuli). A one-sample *t* test revealed that the percentage of participants that yawned when watching the photo stimuli (40%) was significantly higher than that of those who yawned when watching the video stimuli (12.5%), *t* = 2.78, *p* < .01. The Wilcoxon Signed Rank test showed that the frequency of contagious yawning induced by yawning photo stimuli, *Mdn* = 9.11, was significantly higher than that of the yawning video stimuli, *Mdn* = 8.50, *Z* = −2.59, *p* = .01, *r* = −0.41. It is possible that this was due to the presentation strength (20 consecutive yawns without a time gap in the photo stimuli and 10 yawns with a 20-s interval in between yawns). The sporadic nature of yawning in the video stimuli may have caused its decreased effectiveness in provoking contagious yawning in participants. In addition, some yawning moments in the video were covered by hands as this was a natural gesture, which might also contribute to possible reduction when compared with full view of yawning in photo condition.

Table 1 presents a summary of autistic traits, eye-gazing patterns, and sensitivity to emotional expression.

**Table 1.** The Descriptive Statistics of AQ, AOI Fixation in the Video and Photos, and Sensitivity Threshold of Different Expressions.

	M	SD
AOI fixation in video (seconds)		
Eye (total)	19.94	18.34
Mouth (total)	9.87	8.62
Eye (per yawn)	2.10	1.05
Mouth (per yawn)	1.11	.85
Eye-mouth fixation ratio^a^	1.54	2.96
AOI fixation in photo (seconds)		
Eye (total)	28.13	18.34
Mouth (total)	25.52	13.40
Eye (per yawn)	1.73	.89
Mouth (per yawn)	1.47	.71
Eye-mouth fixation ratio^a^	1.48	1.15
Sensitivity threshold^b^		
Happy	1.88	1.43
Angry	1.97	.42
Yawn	2.21	.45

*M* = mean; *SD* = standard deviation; AOI = area of interest.

a The eyes-mouth fixation duration ratio was calculated from the percentage of time fixated at the eye regions and the percentage of time fixated at the mouth region. ^b^The average of individuals' detection threshold.

### Detection Sensitivity Measurements

**Figure 4.**
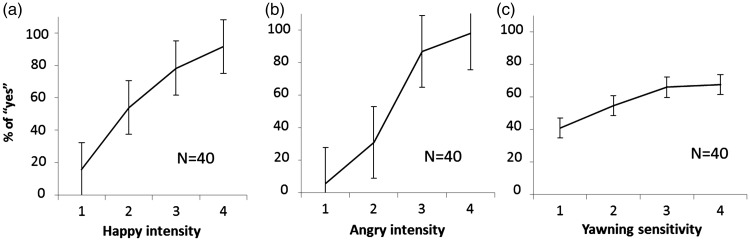
Psychometric curves for happy (a), angry (b), and yawn (c) detection. Error bars represent standard errors.

To derive the detection sensitivity of expression, we defined the threshold as the expressive intensity level at which participants reported detection of an expression half of the time (i.e., 50%). In other words, a higher intensity of expressiveness indicates a lower sensitivity. The psychometric curves for detecting yawning, happiness, and anger are shown in Figure 4.

### Gender, Age, and Contagious Yawning

A recent study reported a female advantage of susceptibility to contagious yawning (Norscia, Demuru, & Palagi, 2016a). Therefore, we examined if there were any gender differences before analyzing the relationship of AQ and contagious yawning. The Poisson regression indicated that there was a significant gender difference in yawning frequency, χ^2^(1, *N* = 40) = 7.96, *p* = .005, e^B ^= .27: Females showed more yawning than male. Moreover, since Bartholomew and Cirulli (2014) found that age is the only variable that contributes to individual variation of yawning susceptibility, we also examined the relationship between age and yawning counts. However, the association between age and contagious yawning was nonsignificant (Appendix A).

**Table 2.** Summary of Poisson Regression Predicting Count of Yawn by Gender and Sensitivity to Yawning.

			95% Wald confidence interval		Hypothesis testing				95% Wald confidence interval	
Variable	β	*SE*	Lower	Upper	Wald χ^2^	*df*	*p*	e^B^	Lower	Upper
(Intercept)	−.60	.39	−1.36	.17	2.34	1	.13	.55	.26	1.18
Gender	−1.30	.46	−2.20	−.40	7.96	1	.005	.27	.11	.67
Sensitivity	1.10	.44	.24	1.95	6.34	1	.012	3.0	1.23	7.06

β = Poisson regression coefficient; SE = standard error; Z = *z*-value; Wald χ^2 ^= Wald chi-square; e^B ^= odd ratios; Sensitivity = perceptual detection sensitivity to yawning.

Gender, and sensitivity were represented as two dummy variables with male, and low sensitivity serving as the reference groups.

### Autistic Traits and Contagious Yawning

We examined the association between autistic traits and contagious yawning using a Poisson regression (Hoogenhout et al., 2013).

Participants were grouped into one of two categories based on their AQ score: Those whose AQ score was higher than the median (i.e., 18) and those whose score was lower than the median. Individuals with higher autistic tendencies (i.e., low AQ score) tended to show less contagious yawning (0.5 for high AQ and 1.31 for low AQ), but the Poisson regression shows that AQ is not significantly associated with count of contagious yawning, χ^2^(1, *N* = 40) = 2.22, *p* = .14, e^B ^= 1.8.

### Detection Sensitivity and Contagious Yawning

We separated participants into two groups, one with high perceptual detection sensitivity (i.e., requiring a lower threshold to detect yawning) and one with low perceptual detection sensitivity to yawning expression based on the median (1.3). We ran a Poisson regression to test whether detection sensitivity to yawning expression (high vs. low) could predict the count of contagious yawning. The result indicated that detection sensitivity to yawning expression (low sensitivity as the reference point) was significantly associated with the count of contagious yawning, χ^2^(1, *N* = 40)=6.34, *p* = .016, e^B ^= 3.00, suggesting that individuals with higher detection sensitivity to yawning expression tended to have more counts of contagious yawning (see Table 2). Even after controlling for gender detection sensitivity to yawning was still significantly associated with the count of contagious yawning (see Table 2). We did not find a significant association between count of contagious yawning and sensitivity to happy expression, χ^2^(1, *N* = 40)=1.53, *p*=.216, e^B ^= 1.63, and sensitivity to angry expression χ^2^(1, *N* = 40)=.14, *p* = .71, e^B ^= 1.15.

### Eye-Gaze Pattern

Participants’ eye-gaze patterns were collected during their observation of yawning stimuli. Results of 11 participants in the video session and 9 participants in the photo session were missing because of technical failure. Therefore, the reported results here only included 29 and 31 participants for the video and photo sessions, respectively. This number is below the average participants in similar eye-tracking studies (*N* = 47 in Armstrong & Olatunji, 2012; *N* = 46 in Usui et al., 2013); as such, we provide these observations as a supplementary result only.

To assess the eye-gaze pattern of the participants, the fixation durations on the AOI (i.e., eyes and mouth region of the actors) during yawning were recorded, and the eye-mouth fixation duration ratio was calculated. We separated participants into two groups (“more at eyes” and “less at eyes”) based on the eye-mouth fixation duration ratio median (1.55 in the video session, 1.32 in the photo session). We examined the relationship between the eye-gaze tendency and autistic traits. As the data for eye-mouth fixation ratio were not normally distributed, a nonparametric analysis, the Mann–Whitney test, was applied. Participants who looked more at the eyes in the video session had significantly lower autistic tendency than those who looked less at the eyes (*U* = 70.5, *p* = .031, *r* = −.38).^1^ However, there was no significance difference in the photo session (*U* = 102.5, *p* = .34, *r* = −.05). We also observed that participants who looked “more at the eyes” in the photo session had significantly higher sensitivity to yawning (i.e., a lower intensity threshold to detect yawning) than the “less at eyes” group (*U* = 44.5, *p* = .001, *r* = −0.55)^2^ but not in the video session (*U* = 87.0, *p* = .432, *r* = −.14). We did not find a similar result for happiness (*U* = 120.5, *p* = .794) or anger (*U* = 83.5, *p* = .097). The effect sizes are −.05 and −.29, respectively.

We did not find evidence from our observations that eye-gaze patterns modulated contagious yawning. The Mann–Whitney test indicated that the urge to yawn of the group looking “more at eyes” was statistically equivalent to the group looking “less at eyes” in both video session, *U* = 85.5, *p* = .099, *r* = −.29 and photo session, *U* = 93.5, *p* = .151, *r* = −05. Again, when comparing the “more at eyes” group with the “less at eyes” group, there was no significant difference for frequency of yawning, χ^2^(1, *N* = 40)=.06, *p* = .81, e^B ^= 1.10.

## Discussion

This study revealed that an individual’s susceptibility to contagious yawning was associated with his or her yawning detection sensitivity. More specifically, people with higher perceptual detection sensitivity to yawning are especially susceptible to contagious yawning. We did not find that autistic tendencies in this nonclinical population were associated with contagious yawning. We found an unexpected gender effect: Females displayed more contagious yawning than males. In summary, gender and yawning detection sensitivity were the two variables associated with individuals’ susceptibility for contagious yawning.

Our hypothesis that perceptual detection sensitivity to yawning expression contributes to the susceptibility of contagious yawning was supported. Although the underlying mechanism remained unclear, sensitivity to yawning could be a possible precursor of contagious yawning and might be facilitated by gazing at the eye region of the inducer. This is in line with the view from the perceptual perspective that sensitivity to others’ expressions (Provine, 1989) and proclivity to look at the eyes (Senju et al., 2009; Usui et al., 2013) play a role in determining the individual’s susceptibility to contagious yawning. These intriguing findings directed the study of contagious yawning from exploring a higher level of processing (e.g., empathy) to a lower level of processing (e.g., eye scanning patterns and perceptual detection sensitivity). Our findings add to the body of research showing that perceptual detection sensitivity to facial expression may alter one’s social processing. For example, Kuusikko et al. (2009) used a computer-based emotional recognition test and asked participants with autism to select the correct upper facial basic emotion for each picture. They found that autistic girls scored lower in recognizing happiness and anger than nonautistic girls. This implies a lower sensitivity to emotional expressions and that more cues may be required to detect an emotion. This impedes their social processing and might lead to inappropriate responses in social interactions. Past studies found that patients with depression also exhibit biased emotion perception in emotional recognition tasks, in which they demonstrated lower sensitivity than controls when asked to distinguish happy from neutral expression (Gur et al., 1992; Mikhailova, Vladimirova, Iznak, Tsusulkovskaya, & Sushko, 1996; Surguladze et al., 2004). This reduced ability could have a significant impact on a person’s social processing, which in turn could affect their own emotion experiences. While earlier research on contagious yawning highlighted its social and empathetic components by showing that poorer social understanding (e.g., in autism, Senju et al., 2007; and schizophrenia, Platek et al., 2003) depleted one’s susceptibility to contagious yawning, here we offered another possible early perceptual origin. Hence, our finding implied that the later processing of the social information delivered by a yawn may be precluded by low sensitivity to yawning and results in a lower susceptibility to contagious yawning.

In addition, our hypothesis that gaze to eye regions enhanced one’s perceptual detection sensitivity to expressions including yawning was partly supported by the positive association between fixation duration to the eye region and perceptual detection sensitivity to yawning. This finding is consistent with a study in which eye-gazing patterns of autistic and nonautistic children were recorded when they viewed morphing facial expressions of six different emotions and labeled the emotions (Bal et al., 2010). Results showed that looking more at the eyes was associated with fewer errors in recognition of disgust and surprise and faster reaction times for recognizing fear but not other emotions. This suggested that attention to the eye region increased sensitivity to emotion recognition, at least for fear, surprise, and disgust. Our study extends this finding beyond emotion recognition to the recognition of yawning.

Our study also reported a female advantage in susceptibility to contagious yawning, which could originate from several possible sources. First, a female advantage in yawning susceptibility could come from a female bias in empathetic ability (for a review, see Christov-Moore et al., 2014), which is known to be related to contagious yawning (Helt et al., 2010; Nahab et al., 2009; Platek et al., 2005; Schürmann et al., 2005; Senju et al., 2007). Females score higher on self-reported empathy questionnaires than males (Rueckert & Naybar, 2008) and show stronger neural activation in empathy-related brain regions such as the amygdala (Schulte-Rüther, Markowitsch, Shah, Fink, & Piefke, 2008). However, in our sample, autistic tendency measured by the AQ did not differ between the two genders. Second, females were reported to excel in emotion recognition tasks (Hall & Matsumoto, 2004; McClure, 2000; Montagne, Kessels, Frigerio, de Haan, & Perrett, 2005; Thompson & Voyer, 2014) and were identified as being better at expressing their own emotions than men. Neuroimaging studies also find that females, but not males, show increased activation of the right inferior frontal cortex, which is known for being involved in emotional contagion (Derntl et al., 2010). In our sampled population, our female participants had marginally significant higher sensitivity than men in detecting happy faces (*p* = .063), but not in angry or yawning faces. Therefore, there was no direct evidence to connect this possibility to our finding. Finally, females are more sensitive to nonverbal cues in social communication than men (Hall & Matsumoto, 2004; McClure, 2000; Montagne et al., 2005; Thompson & Voyer, 2014). It is possible that females are more susceptible to contagious yawning than males due to their higher abilities in understanding others’ intention through nonverbal cues like yawning. We have no direct measurements to test this hypothesis, and this will be a direction for future studies. Norscia and coworkers (2016a) observed the natural occurrence of contagious yawning among 92 nonstranger dyads and found that female participants yawned more frequently than men in response to others’ yawning. As the understanding of gender difference in the contagion effect is still at its infancy, future studies are warranted.

There are at least 15 negative reports on gender differences in contagious yawning, and it is worth noting the differences between them and our study. First, 3 of the 15 studies investigated children with autism (Helt et al., 2010; Senju et al., 2007, 2009; Usui et al., 2013), a population known to have a low yawning frequency (Senju et al., 2007). This may limit any detectable gender effect. Studies with nonclinical populations (e.g., the current study and Norscia et al., 2016a) might offer a bigger observable range. Moreover, a sexually immature group like children is not a good sample group for studying gender differences (Norscia, Demuru, & Palagi, 2016b). Second, 5 of the 15 studies did not include a baseline condition to control for spontaneous yawning (Bartholomew & Cirulli, 2014; Eldakar et al., 2015; Gallup, Church, Miller, Risko, & Kingstone, 2016; Gallup & Eldakar, 2011; Massen, Dusc, Eldakar, & Gallup, 2014). A control condition (i.e., in our case, a smiling video) or a baseline measurement (e.g., spontaneous yawning in Norscia et al., 2016a) offers an assurance that the recorded yawning was not predominantly spontaneous yawning, which is known to have no gender difference (Schino & Aureli, 1989), but contagious yawning which was our central research interest. Lastly, contagious yawning could be significantly diminished by social presence (Gallup et al., 2016), and females are more sensitive to social etiquette (Baron-Cohen, O’Riordan, Jones, & Plaisted, 1999). It is possible that the initial female advantage to yawning susceptibility was counterbalanced by the higher inhibition of yawning behavior in females. Participants in 4 of the 15 studies were aware of experimenters’ observation of their yawning behavior as they viewed the yawning photos directly in front of the experimenter (Eldakar et al., 2015; Gallup & Eldakar, 2011; Gallup & Gallup, 2007; Massen et al., 2014). This makes it possible that the gender difference was absent due to the pressure to inhibit yawning behavior, especially in females, due to the social presence of the experimenters. To reduce participants’ awareness of having their yawning behavior observed, our cover story disguised the real study purpose and a hidden webcam was installed to record their yawning for later analysis. In summary, our control baseline, cover story, and experimental design may offer a more suitable combination to observe a female advantage in susceptibility to contagious yawning.

While a previous study reported that age is the only variable contributing to individual susceptibility to contagious yawning (Bartholomew & Cirulli, 2014), we did not find similar evidence for older individuals being less susceptible to yawning when they watched yawning video clips and still images. Our sample had a relatively narrow age range (i.e., 19–26 years old) compared with Bartholomew and Cirulli’s study (i.e., 18–83 years old), hence, it may not be large enough to reflect similar age effect.

Our finding that participants with more autistic traits have a lower tendency to gaze at eyes adds to recent reports of an association between autistic traits and eye gazing in nonclinical populations. Individuals with more autistic traits (assessed using the Broad Autism Phenotype Questionnaire) tended to look less at the faces of experimenters who asked participants questions (Vabalas & Freeth, 2016) and had shorter and less frequent saccades on the face during a face-to-face interaction with the experimenters (Freeth, Foulsham, & Kingstone, 2013) compared with participants lower in autistic traits. In a recognition task of artificial faces, Davis et al. (2017) discovered that those who had higher AQ scores on the AQ-social subscale tended to look less at eye regions of the facial stimuli. Our results extend the findings to human face photos and videos and indicate that nonclinical individuals lower in autistic traits tend to gaze more at the eyes. This is supplementary to the well-established report of individuals with autism looking less at the eyes (Blair, 2005; Gepner et al., 1996; Golarai et al., 2006).

However, we did not find an association between eye-gaze patterns and contagious yawning. This is possibly because the face studying patterns among the nonclinical population were consistently concentrated on the key features. The aforementioned studies revealed the connection with contagious yawning through comparisons between autistic and nonautistic children (Senju et al., 2009; Usui et al., 2013), which might cover a bigger range of differences.

There are several possible directions for future studies. Despite our efforts in disguising the main purpose of our study and minimizing participants’ awareness of their own yawning, some participants still reported that they suppressed their yawning due to social etiquette. This could potentially affect subjects’ yawning frequency in response to the yawning stimuli. We included a question on the subjective urge to yawn as a supplementary tool for measuring yawning tendency. In addition, a more objective method, such as myography that measures muscle activity, could be helpful to measure subjects’ yawning tendency, including those that are suppressed and undetectable to observation. Moreover, it is worth noting that our yawning stimuli produced a flatter psychometric curve than happy and angry stimuli. All stimuli selected to construct psychometric curves for the three expressions were based on the same intensity rating method, so it is unclear what constituted this difference. While the faces considered as the happiest or angriest in the rating task were always considered as “YES-presence” in the detection ask, and the neutral faces are always considered as “NO-presence” in the detection ask, such connection was less clear in yawning. One possibility is that participants have adopted a different criterion in the Yes-No detection task for yawning from that for happy or angry faces, possibly due to the lack of experience on yawning presence judgment. It is still an unsolved puzzle to us why this occurs, and it may be a possible direction for future study. Lastly, a more extensive screening to exclude participants with clinical conditions and trait-like alexithymia (the lack of own emotion understanding) could be applied in future studies. Individuals with psychiatric disorders such as autism and schizophrenia could have lower susceptibility to contagious yawning, and individuals with alexithymia (which could be measured with the Toronto Alexithymia Scale, Bagby, Parker, & Taylor, 1994; Bird, Press, & Richardson, 2011) may be limited to report accurately on their urge to yawn. Our postexperiment questionnaire showed that our participants were not diagnosed with any psychiatric disorders, and it may be desirable to employ a more extensive pretest screening to rule out factors that could affect sensitivity and susceptibility to yawning in future studies.

## Conclusion

Our study revealed that participants with higher yawning sensitivity (but not emotional sensitivity) are more susceptible to contagious yawning, which adds to the growing literature that suggests perceptual deficits, such as atypical eye-gaze patterns, might contribute to reduced behavioral contagion. In addition, females were found to display more contagious yawning than males, and the underlying mechanism awaits elucidation by future studies. These findings have important theoretical implications for understanding the mechanism of contagious yawning for a nonautistic population. It will be interesting to see whether similar associations exist in a clinical population such as people with autism.

In conclusion, our study offers a working hypothesis for future studies to investigate how the perceptual encoding of yawning interacts with susceptibility to contagious yawning.

## Author Note

Author Meingold H.M. Chan is also affiliated to Department of Human Development and Family Science, Ohio State University, Ohio, USA.

## Notes

1 We also log transformed the eye-mouth ratio for an independent *t* test; the result remained the same, *t*(30) = −2.70, *p* = .01.

2 We also log transformed the eye-mouth ratio for an independent *t* test; the result remained the same, *t*(28) = −2.96, *p* = .006.

## Appendix A. Summary of Poisson regression predicting count of yawn by age.

**Table:**

			95% Wald confidence interval		Hypothesis testing				95% Wald confidence interval	
Variable	β	*SE*	Lower	Upper	*Wald* χ^2^	*df*	*p*	e^B^	Lower	Upper
(Intercept)	−.693	1.00	−2.65	1.27	.48	1	.49	.50	.070	3.55
Age = 19	.442	1.07	−1.65	2.54	.17	1	.68	1.56	.191	12.64
Age = 20	.693	1.07	−1.40	2.79	.42	1	.52	2	.246	16.26
Age = 21	.405	1.15	−1.86	2.67	.12	1	.73	1.50	.156	14.42
Age = 22	.241	1.07	−1.85	2.35	.051	1	.82	1.27	.157	10.34
Age = 23	−.693	1.41	−3.47	2.08	.24	1	.62	.50	.031	7.99
Age = 24	.288	1.22	− 2.11	2.69	.055	1	.81	1.33	.121	14.70
Age = 26	0									

β = Poisson regression coefficient; SE = standard error; *Z* = *z*-value; Wald χ^2 ^= Wald chi-square; e^B ^= odd ratios.
